# American Medical Association

**Published:** 1857-10

**Authors:** 


### American Medical Association.

The Nashville Journal speaks of Dr's. Boring and Means, in an offensive manner, as the "preaching brethren" of the American Medical Association—please allow us to add that if we are correctly informed, there were no less than three other "preaching brethren," who were members of the Association and at the same time members of the Faculty of the Medical Department of the University of Nashville.

We wish it to be distinctly understood that it is not in an offensive way that we allude to this matter—we mean no disrespect to the "preaching brethren" of the *Nashville* Faculty; but we wish to call attention to the low resorts of the Editor of the Nashville Journal to bring into disrepute those he may wish to injure.

In reference to the insinuations of "cut and dried" and "preconcerted" arrangements, we would refer the Editor of that Journal to the *real transactions* of the nominating committee of the American Medical Association, and enquire if some extraordinary influence did not succeed in *instructing* every member of that committee (with one exception) to vote for a *certain* individual for the Presidency? and in addition to this, we would ask is it likely that this *unanimity* would have prevailed without some "cut and dried" or "preconcerted" arrangement? So far as the Georgia delegation is concerned, we shall not hesitate to say that the *instructions* to vote for Dr. Eve were given, in direct and positive opposition to the will of a large minority.

"These explanations are exceedingly unpleasant to us, but the unjustifiable and most extraordinary course" of the Nashville Journal, "has forced upon us the necessity of making them."

